# Exploratory Clinical Study to Evaluate the Efficacy and Safety of Sulfasalazine for Immune Checkpoint Inhibitor (ICI)‐Induced Colitis

**DOI:** 10.1002/jgh3.70365

**Published:** 2026-02-17

**Authors:** Mariko Kobayashi, Takeshi Yamada, Shunsuke Ueyama, Yoshiyuki Yamamoto, Akinori Sugaya, Yoshinori Hiroshima, Takashi Mamiya, Junji Hattori, Shintaro Akiyama, Bryan J. Mathis, Kiichiro Tsuchiya

**Affiliations:** ^1^ Department of Gastroenterology University of Tsukuba Hospital Ibaraki Japan; ^2^ Department of Gastroenterology Tsuchiura Kyodo General Hospital Ibaraki Japan; ^3^ Department of Medical Oncology Ibaraki Prefectural Central Hospital Ibaraki Japan; ^4^ Department of Gastroenterology Hitachinaka General Hospital Ibaraki Japan; ^5^ Department of Gastroenterology Ryugasaki Saiseikai Hospital Ibaraki Japan; ^6^ Department of Cardiovascular Surgery University of Tsukuba Hospital Ibaraki Japan; ^7^ Institute of Medicine, Clinical Research Promotion Group, University of Tsukuba Ibaraki Japan

**Keywords:** adverse effects, immune checkpoint inhibitors, inflammatory bowel disease, sulfasalazine

## Abstract

**Background:**

The first‐choice treatment for immune checkpoint inhibitor (ICI)‐induced colitis is steroids; however, side effects may occur and survival may be reduced. Sulfasalazine (SSZ) is primarily used for inflammatory bowel disease but studies on ICI‐induced colitis are scarce. This study examined the efficacy and safety of SSZ for ICI‐induced colitis while testing SSZ as a steroid‐sparing agent.

**Methods:**

This study (iRECSA) was a single‐arm, multicenter exploratory study from November 2021 to December 2024 that evaluated the efficacy and safety of SSZ for mild‐to‐moderate ICI‐induced colitis. SSZ was given at 4 g/day orally for 2 weeks. The primary outcome was clinical response (a ≥ 1‐point reduction from baseline partial Mayo score or partial Mayo score < 1). Secondary endpoints included clinical responses in the Simple Clinical Colitis Activity Index (SCCAI), Lichtiger Index, and Common Terminology Criteria for Adverse Events (CTCAE) grade, plus SSZ‐related adverse event incidence.

**Results:**

Ten patients were enrolled. The median partial Mayo score decreased from 4 (range 3–5) to 2 (range 0–4), with 80% of patients achieving a clinical response. Similar response rates were observed with the Lichtiger Index and CTCAE grade. Defecation urgency was present in 70% of patients at baseline but resolved in all after treatment. Nine adverse events occurred in six patients; three patients discontinued SSZ because of hypersensitivity‐related adverse events. Three patients required systemic steroids after the study treatment.

**Conclusions:**

This exploratory study suggests that SSZ may be a useful option for mild‐to‐moderate ICI‐induced colitis, but caution is warranted against SSZ‐related hypersensitivity.

**Abbreviations**
5‐ASA: 5‐aminosalicylic acid
ALT: alanine aminotransferase
AST: aspartate aminotransferase
CTCAE: Common Terminology Criteria for Adverse Events
ECOG: Eastern Cooperative Oncology Group
IBD: inflammatory bowel disease
ICI: immune checkpoint inhibitor
ICI‐IA: immune checkpoint inhibitor–associated inflammatory arthritis
irAE: immune‐related adverse event
jRCT: Japan Registry of Clinical Trials
NSAIDs: non‐steroidal anti‐inflammatory drugs
PPI: proton pump inhibitor
PSL: prednisolone
SCCAI: Simple Clinical Colitis Activity Index
SSZ: sulfasalazine
UC: ulcerative colitis
UCEIS: Ulcerative Colitis Endoscopic Index of Severity

## 1 Introduction

Diarrhea and colitis represent clinically important gastrointestinal immune‐related adverse events (irAE) associated with immune checkpoint inhibitor (ICI) therapy. The incidence of ICI‐induced diarrhea has been reported to be 12.1%–13.7% with PD‐1 inhibitors and 30.2%–35.4% with CTLA‐4 inhibitors [1]. Due to the quality of life‐threatening symptoms of ICI‐induced colitis, effective management strategies are clinically important. As ICI‐induced colitis management is similar to inflammatory bowel disease (IBD) (i.e., ulcerative colitis [UC]), the currently recommended initial treatment is steroids. However, high‐dose steroids for long durations during anti‐PD‐1 therapy may impair antitumor immune activity [2], plus generate a surfeit of known side effects [3]. Therefore, a conservative combination of loperamide and hydration has been suggested for patients with diarrhea below grade 2, starting glucocorticoids only if symptoms worsen or persist [4]. In mild cases of UC, mesalamine is first administered, with steroids given only if there is no improvement [5]. However, only few reports have shown that mesalamine is appropriate for persistent, grade 1, ICI‐induced colitis [6, 7].

Sulfasalazine (SSZ) is a synthetic salicylic acid derivative widely used in UC and rheumatic diseases. Upon metabolism by bacteria in the colon, SSZ separates into 5‐aminosalicylic acid (5‐ASA; the active component) and sulfapyridine moieties [8] that putatively mediate the majority of adverse reactions. For UC, reports indicate that SSZ does not differ from 5‐ASA in terms of efficacy or tolerability [9, 10] and a Cochrane systematic review demonstrated no significant difference in remission induction while suggesting superior cost‐effectiveness of SSZ compared with oral 5‐ASA [11]. In Japan, SSZ is also reimbursed for the treatment of non‐specific inflammatory bowel disease, further supporting its clinical feasibility.

Beyond IBD, SSZ has been recommended as an add‐on therapy to corticosteroids or as a steroid‐sparing agent for immune checkpoint inhibitor–associated inflammatory arthritis (ICI‐IA) [12, 13, 14, 15]. Although hypersensitivity reactions have been reported more frequently in ICI‐treated patients and enhanced drug‐specific T‐cell priming against sulfapyridine has been suggested [16], clinical data on SSZ use during ICI therapy remain limited [17], and reports in ICI‐induced colitis are particularly scarce [18]. Therefore, we conducted an exploratory study to evaluate the efficacy and safety of SSZ in ICI‐induced colitis.

## 2 Methods

### 2.1 Study Design and Patient Population

This multicenter single‐arm, exploratory study (iRECSA) was planned to be conducted between November 2021 and September 2025 but was ended on December 31, 2024 due to successfully meeting recruitment goals. The iRECSA study was designed to evaluate the efficacy and safety of SSZ in treating ICI‐induced colitis in cancer patients. Considering feasibility, this study was designed as a pilot study with a sample size of 10 cases to evaluate efficacy and safety. Written, informed consent for research was obtained from each patient prior to the commencement of the study. We conducted the present study in accordance with the Clinical Trials Act in Japan and registered the trial in the Japan Registry of Clinical Trials (jRCTs031210429). The study was performed according to the principles of the Declaration of Helsinki after obtaining approval from the Clinical Research Review Board, University of Tsukuba (Approval #TCRB21‐011).

#### 2.1.1 Inclusion Criteria

- Diarrhea or bloody stools lasting more than 1 week during treatment with ICI or within 3 months of discontinuation, without spontaneous resolution.
- Mayo scores of 3–10 and a Mayo endoscopic subscore > 1
- Age ≥ 20 years.
- Performance status 0–2.
- Written informed consent provided.

#### 2.1.2 Exclusion Criteria

- Colitis diagnosed by lower gastrointestinal endoscopy is clearly due to other diseases.
- History of inflammatory bowel disease.
- Using or planning to use steroids in the week prior to enrollment and in the 2 weeks following enrollment (physiologic replacement dose of up to 20 mg/day of hydrocortisone for adrenal insufficiency and topical or inhaled steroids were acceptable).
- Suspected or confirmed toxic megacolon or gastrointestinal perforation.
- Uncontrolled concomitant irAEs.
- Severe hepatic dysfunction (AST or ALT ≥ 100 IU/L).
- Severe renal dysfunction (serum creatinine ≥ 2.0 mg/dL).
- History of hypersensitivity to sulfa or salicylates.
- Poorly controlled bronchial asthma (asthma attack within 1 month).
- Pregnancy or lactation.
- Any condition deemed inappropriate by the investigator.

### 2.2 Intervention and Study Flowchart

**FIGURE 1:**
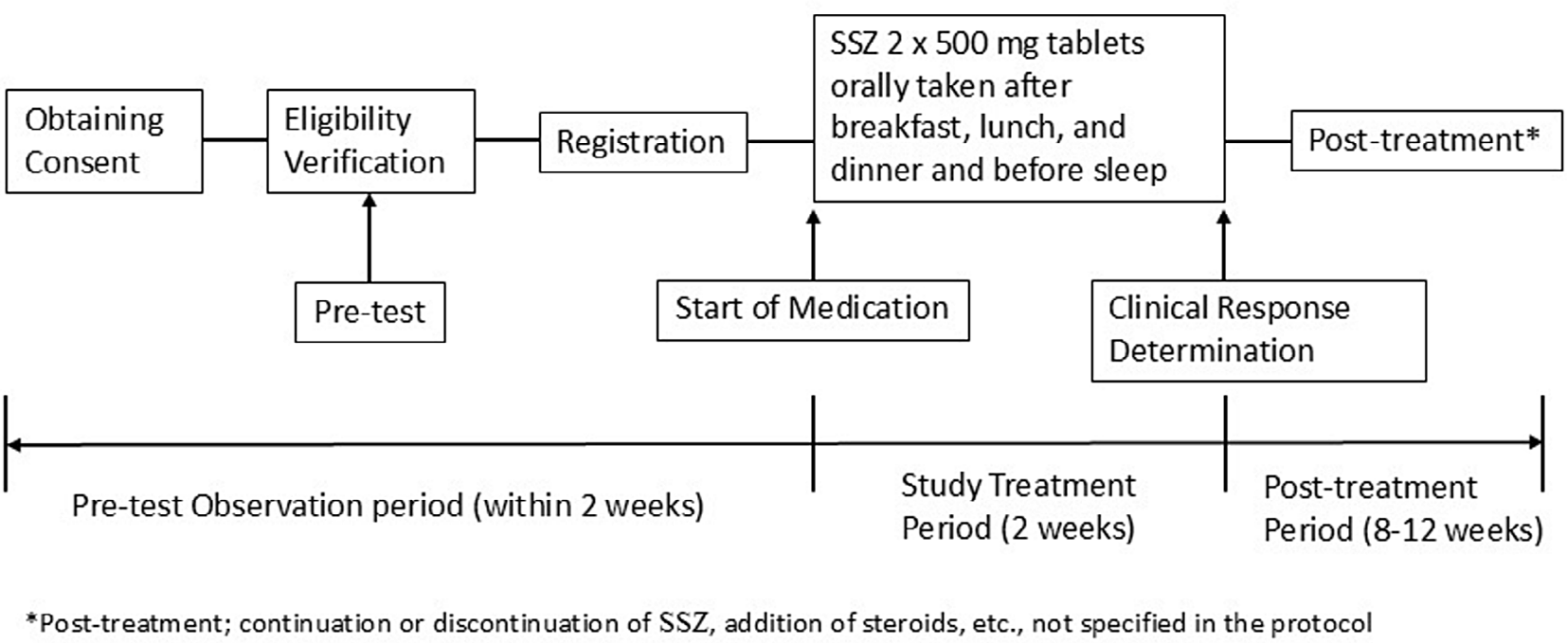
Flowchart of the study.

ICI‐induced colitis was diagnosed based on the presence of symptoms and the absence of a more probable competing diagnosis [19]. Symptoms include diarrhea or bloody stools lasting more than 1 week during treatment with ICI or within 3 months of discontinuation. More probable competing diagnoses included IBD, drug‐induced colitis, ischemic colitis, or infectious colitis. To arrive at diagnoses, we performed interviews, took medical histories, and did CT scans, colonoscopies, histopathological examinations, stool cultures, and *Clostridium difficile* toxin detection testing on all patients. SSZ was given at 4 g/day orally (2 × 500 mg tablets/dose, after breakfast, lunch, and dinner and before bedtime) for 2 weeks as the study treatment period. In the study protocol, initiation at reduced doses (1.5–3 g/day) was permitted when there were concerns regarding potential adverse events or decreased adherence due to pill burden. Dose reductions during treatment of up to 1.5 g/day were also permitted due to physical condition or minor adverse events. Therefore, the dosing strategy was intentionally designed to provide flexibility and to reflect real‐world clinical practice. Note that the use of concomitant steroids (prednisolone, methylprednisolone, etc.), other than as physiologic replacement, was prohibited. The concomitant use of biologics (e.g., infliximab, immunomodulators, immunoglobulin preparations) and 5‐ASA was also prohibited. Interviews about symptoms were given at 1 and 2 weeks after SSZ was started, when it was stopped, and 10 weeks after SSZ was started. At the end of the study, a colonoscopy was not required. A study flowchart is available in Figure 1.

### 2.3 Outcomes

As the Common Terminology Criteria for Adverse Events (CTCAE) diarrhea grade alone is insufficient for evaluating ICI‐induced colitis, evaluation according to IBD criteria is recommended [20]. Thus, in this study, we evaluated clinical symptoms using UC indicators (partial Mayo score [21], Simple Clinical Colitis Activity Index (SCCAI) [22], and Lichtiger Index [23, 24]), as well as the CTCAE. We assessed colonoscopic findings prior to study entry using the Mayo endoscopic subscore and the Ulcerative Colitis Endoscopic Index of Severity (UCEIS), which are both used in UC [25]. In this study, we applied these UC indicators exploratively to more accurately evaluate symptoms related to colitis, such as urgency and bloody stools, which are difficult to capture using the CTCAE. The primary outcome was clinical response, defined as a ≥ 1‐point reduction from the baseline partial Mayo score or a partial Mayo score < 1. Secondary outcomes included clinical response (either a > 1.5‐point reduction from the baseline SCCAI, a ≥ 3‐point reduction from the baseline Lichtiger Index, or a ≥ 1‐point reduction from the baseline CTCAE grade of diarrhea), post‐treatment, percentage of transition to steroid treatment, and SSZ‐related adverse events. For patients who discontinued or withdrew from treatment, efficacy and safety assessments were performed at the time of discontinuation as prespecified in the protocol. In patients who discontinued treatment, the relative dose intensity was calculated based on the treatment period up to discontinuation. All 10 patients who initiated SSZ were included in the analysis, irrespective of subsequent dose modifications.

### 2.4 Adverse Events

Patients were monitored for clinical evidence of SSZ toxicity, including skin disorders, gastrointestinal disorders, hepatotoxicity, myelotoxicity, and pancreatitis. All adverse events were recorded and evaluated by CTCAE v.5.0 guidelines.

### 2.5 Statistical Analysis

Since there are no established agents in the treatment of ICI‐induced colitis prior to steroid administration, we could not establish a comparative control group. Continuous variables are expressed as median and range. The Wilcoxon signed‐rank test was used to compare clinical scores before and after the 2‐week treatment period or at the time of SSZ discontinuation. A two‐sided *P*‐value of < 0.05 was considered statistically significant. All statistical analyses were performed using IBM SPSS Statistics ver. 30.0 (IBM Corp., Armonk, New York, USA).

## 3 Results

### 3.1 Baseline Characteristics

A total of 10 patients were enrolled in the study, with a median age of 59 years (range: 45–82 years) at the time of obtaining consent. Baseline characteristics of the 10 patients are outlined in Table 1. Eight patients were receiving PD‐1/PD‐L1 inhibitor monotherapy while 2 patients were receiving combination therapy of PD‐1 and CTLA‐4 inhibitors. The median time to develop colitis due to ICI was 3.3 months (range: 0.8–22.5 months). All patients had discontinued ICI at the time of enrollment and ICI was not reintroduced in any patient during the study period. At the time of trial participation, 4 patients were taking proton pump inhibitors (PPI) but none were taking non‐steroidal anti‐inflammatory drugs (NSAIDs) or laxatives. Two cases were complicated by cutaneous toxicities and 4 by thyroid dysfunction. Table 2 shows the baseline activity of ICI‐induced colitis. Before treatment, the median partial Mayo score was 4 (range: 3–5), the median Mayo score was 5 (range: 4–6), the median SCCAI was 4.5 (range: 3–7) and the Lichtiger index median score was 7 (range: 5–11). The Mayo endoscopic subscore, which reveals the endoscopic severity associated with UC, was 1 point in 8 patients and 2 points in 2 patients. The UCEIS, which also evaluates endoscopic severity, was scored 1, 2, and 3 in 6, 2, and 2 patients, respectively. The disease extent was left‐sided colon in 2 patients and entire colon in 8 patients. All 10 cases had at least one active histological inflammation feature, which included neutrophilic or eosinophilic infiltrate, cryptitis, crypt abscess, and/or apoptosis [26]. In all cases, CT examination revealed no clear findings suggestive of enteritis, such as edematous wall thickening of the small intestine.

**TABLE 1:** Characteristics of patients with ICI‐induced colitis.

Patient characteristics	*N* = 10
Age (year)	
40–49	1
50–59	2
60–69	3
70–79	3
80+	1
Gender	
Female	4
Male	6
Original tumor	
Esophageal cancer	2
Gastric cancer	2
Hepatocellular carcinoma	2
Lung cancer	1
Melanoma	1
Uterine cervical cancer	1
Carcinoma of unknown primary	1
ECOG performance status	
0	3
1	4
2	3
Medications	
Proton pump inhibitors	4
Potassium‐competitive acid blockers	2
Non‐steroidal anti‐inflammatory drugs	0
Antibiotics	0
Laxatives	0
Antiplatelet drugs	0
Anticoagulants	2
Immunotherapy	
Nivolumab	5
Nivolumab+ipilimumab	1
Pembrolizumab	2
Atezolizumab	1
Durvalumab+tremelimumab	1
Duration from starting immunotherapy to occurrence of ICI‐induced colitis	
1 month	4
2 months	0
3 months	2
4 months	1
5 months	1
6 months+	2
Co‐existence with other immune‐related adverse events	
Cutaneous toxicities	2
Thyroid dysfunction	4

*Note:* Data are presented as *n*.

Abbreviations: ECOG, Eastern Cooperative Oncology Group; ICI, Immune checkpoint inhibitor.

**TABLE 2:** Baseline activity of ICI‐induced colitis.

	*N* = 10
Partial Mayo Score	
3	4
4	3
5	3
Mayo Score	
4	3
5	3
6	4
SCCAI	
3	1
4	4
5	2
6	2
7	1
Lichtiger Index	
5	1
6	3
7	1
8	2
9	2
11	1
CTCAE	
2	4
3	6
Mayo Endoscopic Subscore	
1	8
2	2
UCEIS	
1	6
2	2
3	2
Disease extent	
Left‐sided colitis	2
Pancolitis	8
Histological findings^a^	
Crypt apoptosis	4
Crypt abscess	4
Neutrophilic or eosinophilic infiltrate	8

*Note:* Data are presented as *n*.

Abbreviations: CTCAE, Common Terminology Criteria for Adverse Events; ICI, Immune checkpoint inhibitor; SCCAI, Simple Clinical Colitis Activity Index; UCEIS, Ulcerative Colitis Endoscopic Index of Severity.

^a^ The patient count has duplicates.

### 3.2 Clinical Efficacy

The initial daily SSZ dose was 4 g/day in 4 patients, 3 g/day in 4 patients, and 1.5 g/day in 2 patients in light of physical discomfort caused by tumor or anticancer drug treatment. The median relative dose intensity among all 10 patients was 74%. Among 10 patients, 5 completed the scheduled treatment, but 5 discontinued it. The reasons for discontinuation were adverse events due to SSZ in 3 patients, complications from other irAE in 1 patient, and a worsening general condition due to cancer and sepsis in 1 patient.

**FIGURE 2:**
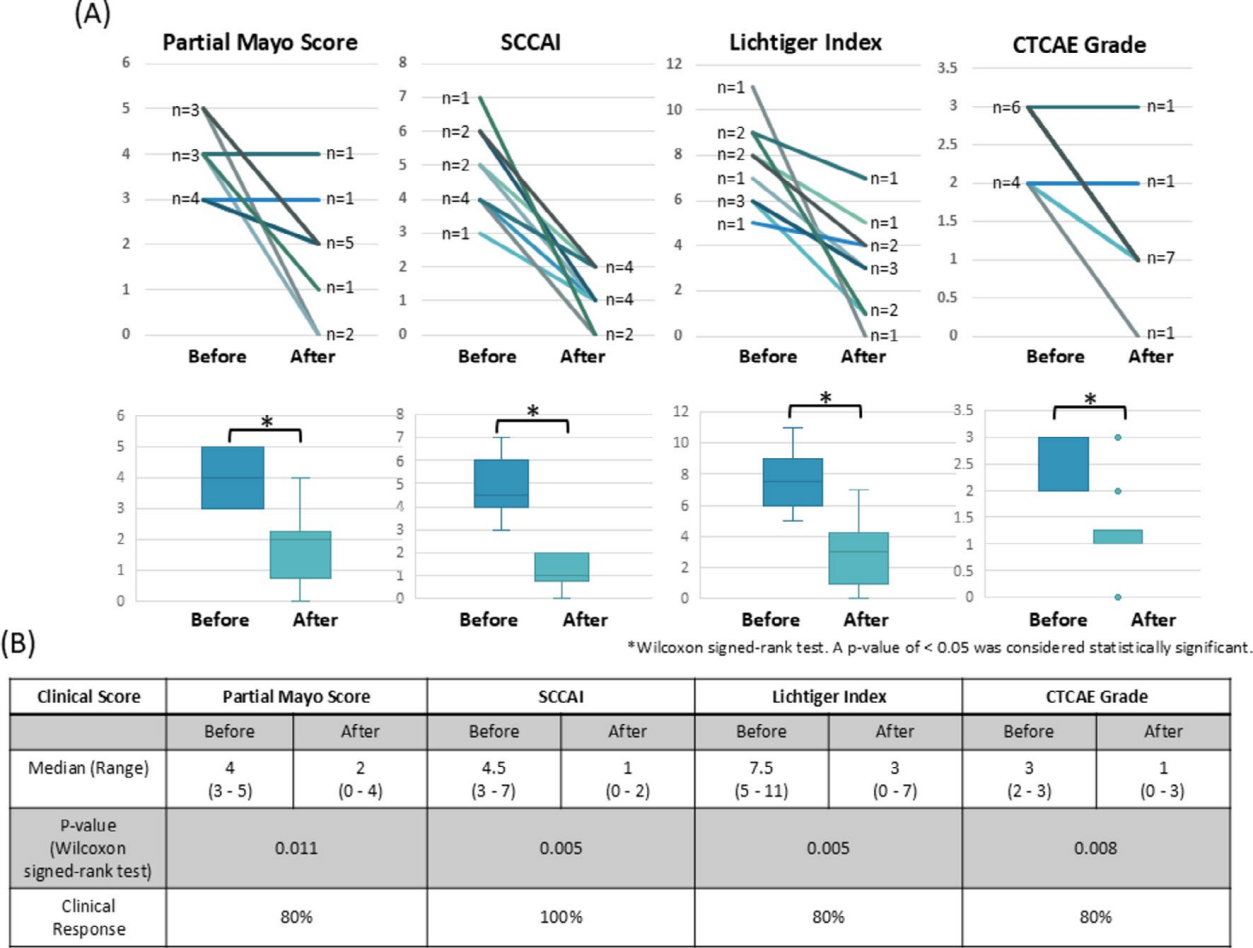
The efficacy of sulfasalazine (SSZ) for ICI‐induced colitis. (A) Changes in the partial Mayo score, Simple Clinical Colitis Activity Index (SCCAI), Lichtiger index, and Common Terminology Criteria for Adverse Events (CTCAE) before and after the administration of SSZ in 10 patients. Data are presented as individual patient trajectories (spaghetti plots) and box‐and‐whisker plots superimposed with individual patient trajectories (*n* = 10). In the box plots, the center line represents the median, the box limits indicate the interquartile range (25th to 75th percentiles), and the whiskers extend to the minimum and maximum values. Each line represents an individual patient's clinical course. Statistical significance was determined using the Wilcoxon signed‐rank test. A two‐sided *p*‐value of < 0.05 was considered statistically significant. (B) Median score for each clinical score before and after SSZ administration and percentage of patients achieving clinical response as assessed by each score. Clinical response was examined in 10 patients using clinical scores after 2 weeks of SSZ administration or at the time of discontinuation. Clinical response was defined as a ≥ 1‐point reduction from the baseline partial Mayo score or a partial Mayo score < 1, a > 1.5‐point reduction from the baseline SCCAI, a ≥ 3‐point reduction from the baseline Lichtiger Index, or a ≥ 1‐point reduction from the baseline CTCAE grade of diarrhea.

Before SSZ administration, the partial Mayo scores were 3 points in 4 patients, 4 points in 3 patients, and 5 points in 3 patients. Following SSZ administration, 2 patients had 0 points, 1 patient had 1 point, 5 patients had 2 points, 1 patient had 3 points, and 1 patient had 4 points. The median partial Mayo score before SSZ was 4 (range 3–5), but decreased to 2 (range 0–4) after SSZ administration (*p* = 0.011). Eight of 10 patients (80%) achieved a clinical response as defined by partial Mayo score at 2 weeks after the first dose or at the time of discontinuation of SSZ. Among the two patients without a decrease in partial Mayo score, the initial dose and relative dose intensity were 4 g/day and 100%, and 3 g/day and 75%, respectively, both greater than the cohort median. Accordingly, no clear association was observed between SSZ dose or relative dose intensity and clinical efficacy. The median SCCAI before SSZ administration was 4.5 (range 3–7) and 1 (range 0–2) after (*p* = 0.005), indicating a clinical response in all 10 patients (100%). Similarly, the median Lichtiger index and CTCAE grade before SSZ administration were 7.5 (range 5–11) and 3 (range 2–3), respectively; after SSZ administration, these results were 3 (range 0–7) (*p* = 0.005) and 1 (range 0–3) (*p* = 0.008), respectively. Clinical response was obtained in 8 of 10 patients (80%) when assessed by both the Lichtiger index and CTCAE grade. Figure 2 shows the change in scores for each indicator before and after SSZ administration.

**FIGURE 3:**
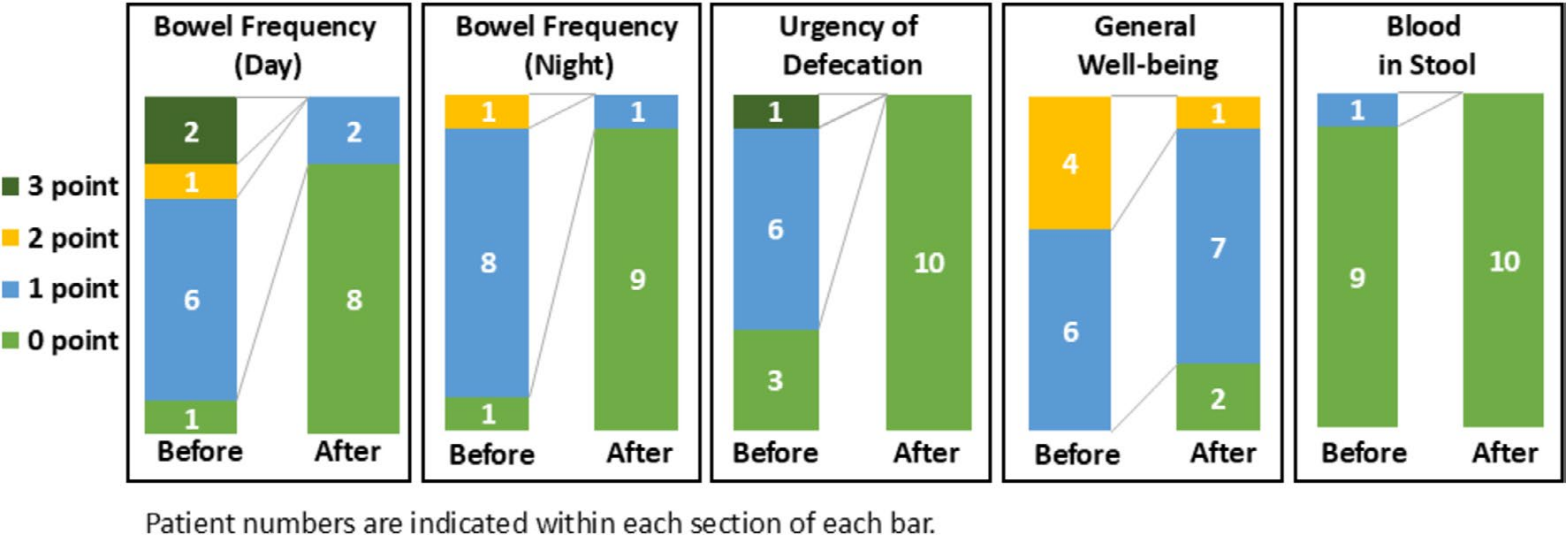
Changes in subscore of the Simple Clinical Colitis Activity Index (SCCAI) before and after the administration of sulfasalazine (SSZ) in 10 patients.

An analysis of the SCCAI subscores revealed that the proportion of patients with a daytime defecation score of 0 improved from 10% to 80%. Similarly, the percentage of patients scoring 0 points for nighttime defecation improved from 10% to 90%. Additionally, 70% of patients reported urgency of defecation before SSZ administration; however, after SSZ administration, defecation urgency disappeared in all patients. Figure 3 shows the change in each SCCAI subscore.

Following the 2‐week medication period, SSZ was continued in 2 out of 10 patients while prednisolone (PSL) was introduced in 3. One patient was started on PSL because treatment for ICI‐induced colitis was interrupted due to an allergy to SSZ, while another was started on PSL because diarrhea improved with SSZ but flared up 2 weeks after treatment ended. Yet another was started on PSL because of complications from ICI‐induced pancreatitis. Five patients were not given any further treatment. Regardless of SSZ discontinuation or PSL administration, after 10 study weeks, ICI‐induced colitis improved in 9 patients (3 with 1 point and 6 with 0 points on the partial Mayo score and 4 with 1 point and 5 with 0 points on the SCCAI), except for 1 patient who died due to worsening cancer. There were no cases of prolonged adverse events from SSZ or delayed steroid induction that led to severe disease or the need for infliximab induction.

### 3.3 Adverse Events

A total of 9 adverse events to SSZ were observed in 6 patients. All adverse events related to SSZ were categorized as grade 2 or less, according to CTCAE version 5.0. Three total patients developed anorexia for which SSZ was decreased in 1 case and continued in the other 2. SSZ was initiated at reduced doses in 6 cases. Anorexia occurred in 2 of the 4 patients who started at the normal dose but occurred in only 1 of the 6 reduced‐dose patients. Skin rash occurred in 4 patients, in which 2 experienced complications of allergic reactions, including fever and joint pain. While the onset of anorexia sometimes occurred relatively early (Days 3, 5, and 11 of SSZ administration), skin rashes and allergies appeared only after Day 9. All patients who experienced these hypersensitivity reactions were treated with PD‐1/PD‐L1 monotherapy. SSZ was discontinued in 2 patients with grade 1 allergies (on Days 11 and 13, respectively) and 1 patient with a grade 2 rash on Day 11. All SSZ‐related adverse events were promptly resolved after cessation of SSZ. Among the 4 patients who initiated SSZ at 4 g/day, all developed appetite loss or hypersensitivity reactions. No SSZ‐related adverse events were observed in 2 of the 4 patients who started at 3 g/day and in both patients who started at 1.5 g/day. These adverse events and SSZ dosage are summarized in Table 3.

**TABLE 3:** Adverse events occurring during the study period.

Adverse events	CTCAE Grade	Onset date	Relevance to SSZ	SSZ initial dose	SSZ relative dose intensity^a^	Action taken with SSZ	SSZ discontinuation date	Treatment after SSZ discontinuation
Anorexia	Grade 2	Day 3	Yes	4 g	27%	Reduced		
Anorexia	Grade 2	Day 5	Yes	4 g	100%	Continued		
Anorexia and Rash	Grade 2 and Grade 1	Day 11	Yes	3 g	75%	Continued		
Rash and Allergy	Grade 1 and Grade1	Day 10	Yes	4 g	100%	Discontinued	Day 13	PSL
Rash and Allergy	Grade 1 and Grade 1	Day 9	Yes	4 g	73%	Discontinued	Day 11	No
Rash	Grade 2	Day 11	Yes	3 g	73%	Discontinued	Day 11	No
Pancreatitis	Grade 3	Day 9	No	3 g	71%	Discontinued	Day 9	PSL
Elevated Creatine Kinase	Grade 1	Day 7	No	1.5 g	41%	Continued		
Sepsis^b^	Grade 5	Day 4	No	3 g	75%	Discontinued	Day 6	No

Abbreviations: CTCAE, Common Terminology Criteria for Adverse Events; PSL, prednisolone; SSZ; sulfasalazine.

^a^ The relative dose intensity of the SSZ in discontinuation cases was calculated based on the period up to discontinuation.

^b^ This patient succumbed to severe sepsis and progressive cancer.

Meanwhile, three adverse events unrelated to SSZ occurred: one case each of ICI‐induced pancreatitis, elevated creatine kinase, and serious sepsis. Regarding pancreatitis, the patient complained of back pain 8 days after starting SSZ. The serum amylase level was elevated to 213 U/L, and a CT scan revealed pancreatic enlargement and increased density of the surrounding adipose tissue. After PSL was administered, the pancreatitis improved rapidly. Table 3 summarizes all adverse events that occurred during the study period.

## 4 Discussion

This is the first report on the potential of steroid sparing in ICI‐induced colitis with SSZ, which was shown to be effective in mild‐to‐moderate cases (Mayo scores of 3–10 and a Mayo endoscopic subscore > 1). In our study, 8 out of 10 patients with mild‐to‐moderate ICI‐induced colitis responded clinically to treatment with SSZ. Furthermore, when evaluated using the SCCAI, all patients showed improvement in colitis symptoms.

In evaluating ICI‐induced colitis, the CTCAE alone is inadequate for assessing severity and predicting response to steroid therapy since the CTCAE grade is defined only by the number of diarrhea episodes [20]. Conversely, the Mayo score (used to assess disease activity in UC) and the UCEIS (used for endoscopic classification) have been shown to be useful [20]. Additionally, for the assessment of clinical UC symptoms, the SCCAI and partial Mayo score were demonstrated to show the best validity and responsiveness [5]. In particular, the SCCAI includes an item to assess the urgency of defecation, which is the most frequent and disruptive symptom that affects quality of life and clinical outcomes in patients with UC [27, 28]. Although no studies have examined bowel urgency in ICI‐induced colitis, we believe it to be as detrimental to quality of life as UC. Therefore, we evaluated the urgency of defecation in ICI‐induced colitis using the SCCAI. Additionally, the SCCAI includes items for extracolonic features, but these were excluded because they are not relevant to ICI‐induced colitis. Finally, as the Lichtiger index also provides a more detailed picture of the disease state, we therefore evaluated the efficacy of SSZ by combining CTCAE grade, the partial Mayo score, the SCCAI, and the Lichtiger index. However, these UC indicators remain unvalidated in ICI‐induced colitis and are not mentioned in the guidelines. Thus, the results of this study should be interpreted accordingly.

Of note, the rate of clinical improvement was 80% when assessed by the partial Mayo score, CTCAE, and the Lichtiger index, whereas it reached 100% when assessed by the SCCAI. These indices differ in the symptom domains they capture. Because only one patient presented with overt rectal bleeding, scores in which rectal bleeding contributes substantially (such as the partial Mayo score) may underestimate symptom improvement. In contrast, the SCCAI and Lichtiger index more sensitively reflect urgency and nighttime defecation frequency, which are closely related to quality of life. Consistent with this, the proportion of patients experiencing urgency of defecation markedly decreased from 70% to 0%, indicating that SSZ was associated with not only stool frequency but also general condition and quality of life. Therefore, the discrepancy among the scores likely reflects differences in symptom coverage rather than true inconsistency in treatment response.

On the other hand, 2 patients who did not show clinical improvement according to the partial Mayo score had an insufficient reduction in the frequency of diarrhea. Both patients received PD‐1/PD‐L1 inhibitor monotherapy, had a UCEIS score of 1 in the entire colon, and showed no signs of erosion or ulceration of the colonic mucosa. Their partial Mayo scores were 4 and 5 before treatment, indicating that the severity of their disease was not high. Therefore, it is difficult to identify a patient profile less likely to respond to SSZ, given that the differences from other cases that have been successfully treated with SSZ are unclear.

For patients with mildly active UC, the guidelines recommend 5‐ASA therapy alone for inducing remission [5]. However, the guidelines for ICI‐induced colitis indicate that steroids would be the first‐choice treatment if ICI withdrawal does not improve the patient's condition [19, 29]. Nevertheless, caution is warranted regarding the side effects of steroids and their potential impact on cancer prognosis. Several studies suggest that high‐dose steroids administered for long periods during anti‐PD‐1 therapy may worsen prognosis by suppressing antitumor immune activity [2, 30, 31]. Therefore, we believe that some cases may potentially benefit from SSZ before steroid administration is considered. Of course, non‐response or exacerbation cases make prompt introduction of steroids essential but careful monitoring is necessary.

In the present study, hypersensitivity‐related adverse events were relatively frequent. Skin rashes were observed in 4 of 10 patients, and 2 developed allergic reactions such as fever or joint pain. This may indicate a significant intolerance burden in patients with ICI‐induced colitis who are treated with SSZ. In contrast, the safety profile of SSZ in UC has been well characterized. In UC, adverse events occurred in 29% of patients treated with SSZ, and treatment discontinuation due to adverse events was reported in 13% [11]. Importantly, hypersensitivity reactions represented only a proportion of these adverse events in UC populations. In rheumatic diseases, hypersensitivity reactions occurred at a rate of 8.8% with SSZ [32]. These findings suggest that the incidence of allergic reactions in this study may be higher than that reported in other diseases.

The discrepancy between the established safety profile of SSZ and the higher frequency of hypersensitivity reactions observed in this study suggests that disease context and concomitant immune modulation may play a critical role. Hypersensitivity reactions may be due to potent antimicrobial activity or T cell‐mediated response to sulfur‐containing moieties that are well‐reported to cause frequent adverse events in some partial population of autoimmune and arthritis cases [33]. In particular, immune activation induced by PD‐1 blockade may enhance susceptibility to SSZ intolerance through immune‐mediated hypersensitivity mechanisms. This interpretation is consistent with prior reports describing increased hypersensitivity reactions to SSZ in patients receiving anti–PD‐1 immune checkpoint inhibitors [17]. Perturbation of drug‐specific T‐cell priming may also contribute to an increased incidence of hypersensitivity [16]. Thus, the higher incidence of allergic reactions in this study is more likely attributable to ICI‐associated immune dysregulation rather than to the baseline tolerability of SSZ observed in UC. However, from a practical standpoint, SSZ may remain clinically relevant. In Japan, SSZ is covered by insurance for nonspecific colitis, facilitating its use in routine clinical practice. In addition, a Cochrane systematic review highlighted the cost‐effectiveness of SSZ, and confirmed that its efficacy for induction of remission in UC is equivalent to that of 5‐ASA [11]. These characteristics support consideration of SSZ as a therapeutic option in ICI‐induced colitis while taking hypersensitivity reactions into account.

As for other adverse events, 3 of 10 patients developed anorexia. These adverse events may be related to the starting dose of SSZ. In fact, adverse events occurred in all 4 cases that began with a 4 g/day dose of SSZ, but only 2 cases experienced adverse events among the six cases that began dose reduction. In spite of the reduction, clinical response, as measured by the partial Mayo Score, was obtained in 5 of the 6 dose‐reduced patients (83%). In this context, although a clear dose response relationship for efficacy was not demonstrated, SSZ‐related adverse events appeared to be more frequent among patients who initiated treatment at 4 g/day compared with those who started at lower doses. These findings suggest that the optimal SSZ dose that balances clinical benefit and tolerability has yet to be determined and warrants further investigation in larger cohorts. In all cases, all adverse events induced by SSZ resolved promptly after administration ceased and it was relatively safe in patients who did not have initial hypersensitivity reactions.

On the other hand, only 1 of the 10 patients developed ICI‐induced pancreatitis during SSZ administration that necessitated study termination and steroid therapy. Although the possibility of SSZ‐induced pancreatitis cannot be completely ruled out, its frequency is reported to be lower than that of 5‐ASA. Pancreatitis was reported seven times more frequently with mesalazine (7.5 per million prescriptions) than with SSZ (1.1 per million prescriptions) (odds ratio, 7.0; 95% confidence interval, 2.6–18.6; *p* < 0.001) [10]. Furthermore, since the pancreatitis improved rapidly with steroid treatment and steroids are not generally considered a standard treatment for drug‐induced pancreatitis, we believe the pancreatitis was caused by ICI. SSZ administration was discontinued when steroids were initiated; however, 3 months later, pancreatic enzymes rose again as the steroids were tapered. Improvement was achieved by re‐escalating the steroids. This episode also supports the conclusion that the pancreatitis was ICI‐induced. Since irAEs appear in multiple organs at various time points, constant vigilance is required when administering SSZ for ICI‐induced colitis, but it is important to note that no study patient experienced delayed steroid treatment or severe disease as a result of participation.

We must acknowledge limitations of our study, which include the small number of patients, the imbalance in underlying tumor types and ICI treatment regimens, high variability in SSZ dosage, absence of systematic small‐bowel evaluation, and a large number of discontinuations. Additionally, because this study lacked a comparator group, we cannot exclude the possibility that the observed clinical improvement reflected the natural history of ICI‐induced colitis rather than a direct effect of SSZ. Although we included only patients with symptoms persisting for more than 1 week despite supportive care and ICI interruption, spontaneous improvement remains possible; therefore, the results should be interpreted with caution. Baseline concomitant medications may also influence diarrhea. Four patients were receiving PPI whereas none were taking NSAIDs, antibiotics, or laxatives. Although PPIs have been associated with collagenous colitis, neither the characteristic “cat scratch” endoscopic appearance nor a subepithelial collagen band on histology was observed in our cohort. Therefore, the contribution of PPIs to diarrhea in these patients is considered unlikely.

Furthermore, recent clinical trials in UC generally adopt more stringent response criteria, such as a decrease of ≥ 2 points and ≥ 30% from baseline and/or a rectal bleeding subscore of 0–1 in partial Mayo score. In contrast, our study used more lenient criteria because most enrollees had mild‐to‐moderate disease with low baseline partial Mayo scores. In these patients, stringent thresholds would have lacked sensitivity to detect early or modest clinical improvement. In addition, our report illustrates the primary difference between IBD and ICI‐induced colitis, namely the presence of cancer‐induced inflammation and poor general condition. In fact, the general condition items included in the partial Mayo score and SCCAI often did not reach 0 points due to deterioration from cancer even if the evaluation criteria for UC included general condition. Taken together, our limitations and differences between IBD and ICI‐induced colitis indicate that disease activity scores originally developed for UC may not fully capture the clinical profile of ICI‐induced colitis, and indicate the need for an optimal evaluation method for ICI‐induced colitis.

The lack of colonoscopic evaluation following SSZ administration may also be a weak point in this study since, in IBD, long‐term therapeutic targets include healing of the mucosa [34]. However, in ICI‐induced colitis, the primary treatment objective differs. The focus is on rapidly controlling symptoms and preventing severe complications given the patients' underlying malignancy and systemic fragility. Unlike in IBD, mucosal healing is neither a validated nor a prioritized endpoint in ICI‐induced colitis. Because invasive procedures, such as follow‐up colonoscopies, may not be appropriate for this fragile population, we removed post‐treatment colonoscopy as a secondary outcome. C‐reactive protein, leucine‐rich alpha‐2‐glycoprotein, and blood sedimentation rate, which are used to evaluate inflammation in IBD, were not examined in this study because some cases would have had high values due to systemic inflammation caused by cancer and not colitis.

In conclusion, SSZ was administered to 10 patients with mild‐to‐moderate ICI‐induced colitis and the clinical improvement rate was 80%. SSZ treatment was associated with clinical improvement in most patients and these findings suggest that SSZ may be a potential steroid‐sparing option. In addition, SSZ administration was associated with bowel urgency and nocturnal defecation which affect quality of life. However, this study was an exploratory single‐arm trial with a small sample size. Only 10 patients were included, SSZ dosing ranged from 1.5 to 4 g/day, treatment discontinuation was relatively frequent, and 3 patients eventually required prednisolone. In addition, the study lacked a control group and robust historical comparators. Consequently, the extent of any true steroid‐sparing effect could not be reliably quantified. Another important consideration is safety. Immune activation induced by ICIs may theoretically increase susceptibility to drug hypersensitivity, and SSZ‐related hypersensitivity reactions could potentially occur more frequently in this setting. Therefore, close monitoring for adverse events, including hypersensitivity reactions, is essential when SSZ is used in patients receiving ICIs.

Taken together, SSZ may be best regarded at present as a potentially useful therapeutic option for patients with mild‐to‐moderate ICI‐induced colitis. Further adequately powered, controlled, comparative trials are needed to clarify its efficacy, optimal dose, safety profile, and its role in minimizing systemic steroid use.

## Funding

This work was supported by Grant for Implementation of Advanced Medicine (GIAM) of the University of Tsukuba Hospital.

## Ethics Statement

This study was approved by the Clinical Research Review Board, University of Tsukuba (Approval #TCRB21‐011) and was conducted in accordance with the principles of the Declaration of Helsinki. Written informed consent was obtained from all participants prior to their inclusion in the study.

## Conflicts of Interest

We disclose conflicts of interest related to the treatment of ICI‐induced colitis, specifically concerning pharmaceutical companies that manufacture and/or market in Japan both branded and generic formulations of SSZ and PSL, as well as infliximab and its biosimilars. The details are as follows:

- Mariko Kobayashi has received grants from Asahi Kasei Pharma Corporation and honoraria for lectures from Takeda Pharmaceutical Company Limited.
- Takeshi Yamada has received grants from Asahi Kasei Pharma Corporation and honoraria for lectures from Takeda Pharmaceutical Company Limited.
- Shunsuke Ueyama has received honoraria for lectures from Takeda Pharmaceutical Company Limited.
- Yoshiyuki Yamamoto has received grants from Asahi Kasei Pharma Corporation and honoraria for lectures from Takeda Pharmaceutical Company Limited and Asahi Kasei Pharma Corporation.
- Akinori Sugaya, Junji Hattori, and Bryan J. Mathis have no conflicts of interest to disclose.
- Yoshinori Hiroshima has received honoraria for lectures from Takeda Pharmaceutical Company Limited, Mitsubishi Tanabe Pharma Corporation, Asahi Kasei Pharma Corporation, and PFIZER JAPAN INC.
- Takashi Mamiya has received honoraria for lectures from Viatris Pharmaceuticals Japan G.K., and Asahi Kasei Pharma Corporation.
- Shintaro Akiyama has received honoraria for lectures from Takeda Pharmaceutical Company Limited, Mitsubishi Tanabe Pharma Corporation, Celltrion Healthcare Japan K.K., Pfizer Japan Inc., Kyorin Pharmaceutical Co. Ltd., and Viatris Pharmaceuticals Japan G.K.
- Kiichiro Tsuchiya has received honoraria for lectures from Takeda Pharmaceutical Company Limited, Mitsubishi Tanabe Pharma Corporation, Celltrion Healthcare Japan K.K., Pfizer Japan Inc., Kyorin Pharmaceutical Co. Ltd., and Viatris Pharmaceuticals Japan G.K.

## Data Availability

The datasets used in this study are not available for sharing, as the informed consent obtained from participants does not include provisions for data sharing with third parties.
